# Carceral tropes in prison-themed escape rooms in Canada, the United States, Australia, and Aotearoa New Zealand

**DOI:** 10.1177/26326663261478660

**Published:** 2026-08-30

**Authors:** Kevin Walby, Linda Mussell, Justin Piché

**Affiliations:** 1 Department of Criminal Justice, 8665 University of Winnipeg, Winnipeg, Manitoba, Canada; 2 Department of Political Science and International Relations, 2496 University of Canterbury, Christchurch, New Zealand; 3 Department of Criminology, 6363 University of Ottawa, Ottawa, Ontario, Canada

**Keywords:** Prisons, escape rooms, commodification, penal spectatorship, carceral tropes, Canada, Australia, Aotearoa New Zealand, United States

## Abstract

Escape rooms have grown in popularity across the world, with a number of these sites established in decommissioned sites of confinement or operating in purpose built faux-prisons. Drawing on fieldwork in prison-themed escape rooms across Canada, the United States, Australia, and Aotearoa New Zealand, this article explores how these tourism and leisure sites contribute to meaning-making vis-à-vis imprisonment and punishment. Beyond examining how these games allow game players to consume punishment at a safe distance from the realities of imprisonment, we show how these venues of penal spectatorship commodify major elements of penality, notably by using wrongful imprisonment and the prospect of death behind bars as carceral tropes. This process of commodification distorts and skews the realities of these phenomena, reinforcing hegemonic views of the punitive injustice apparatus. While the prison escape room experience is meant to entertain and though some sites offer surface-level criticisms of penality, our findings show how these sites reproduce carceral logics that sustain imprisonment as a central practice of exclusion in contemporary times.

## Introduction

The cell bars appear slightly rusty and the toilet has no seat. Emanating from a speaker overhead are random commands from prison officers. Seemingly on repeat, there are the sounds of muffled shouts and metal clanging on metal. The cell keys hang on a wall just outside your reach. A clock is counting down. Despite all the carceral sounds and the contrived sense of being trapped in a cell, this is not a penitentiary, prison or jail, but an escape room. As popular forms of tourism and leisure, escape rooms have expanded across the world over the last twenty years. A number of these sites are erected in decommissioned sites of confinement such as closed jails, prisons, penitentiaries, and police lock-ups that are now operated as heritage sites by government and non-profit agencies or by corporate entities (also see Stuit, 2020a). There are also numerous faux-prison escape rooms, which are commercial sites that simulate a prison environment and may use elements like bars, cell doors, and sinks from decommissioned sites of confinement (Mussell et al., 2023).

While there is a body of literature that focuses on the value of escape rooms as an educational or team-building tool (e.g., Gordon et al., 2019) and tourism attraction (e.g., Pakhalov and Roxhkova, 2020), there is little research examining prison-themed escape rooms and the meanings of penality conveyed therein (but see Mussell et al., 2023; Stuit, 2020a). In this article, we explore what these spaces communicate and fail to convey about imprisonment, along with the extent that these sites raise awareness or reproduce dominant understandings of the complexities of the punitive injustice apparatus. Our work is situated at the intersection of cultural studies of incarceration (Lynch, 2000; Wright, 2000) with focus on representations of imprisonment, as well as critical museum and tourism studies (Knudsen et al., 2016; Stone, 2018) that assess the commodification of penal culture. The objective of our research is to investigate sites of popular culture and representations that communicate stereotypes and metaphors about imprisoned people (e.g., imprisoned people lack intelligence, are untrustworthy and are dangerous) and prisons in our society (e.g., that such institutions are natural and necessary).

Drawing on fieldwork conducted at 31 prison-themed escape rooms across Canada, the United States, Australia, and Aotearoa New Zealand, this article explores how these sites contribute to meaning-making vis-à-vis imprisonment and punishment. Beyond examining how these games allow game players to consume punishment at a safe distance from the realities of imprisonment, we show how these venues of penal spectatorship (Brown, 2009) cash in by commodifying elements of incarceration and penality, notably in prison escape games oriented around two key tropes which are used at the game sites: wrongful imprisonment and death behind bars. This use of carceral tropes – a term we adopt from rhetoric and communication studies to mean stereotypical ideas that underpin the discourse of carcerality (Ho, 2022) – distorts and skews the realities of these phenomena. Such tropes, conveyed through metaphor, metonymy, synecdoche, and irony, reinforce hegemonic views of the punitive injustice apparatus. We show that the escape room prison as spectacle obscures material and specifically Indigenous experiences of incarceration. While the prison escape room experience is meant to entertain and though some sites offer surface-level criticisms of penality, we argue these sites reproduce carceral logics that sustain imprisonment as a central practice of exclusion today.

## Locating prison-themed escape rooms

Escape rooms are in-person or virtual games where teams solve puzzles, locate clues, and accomplish tasks with the goal of escaping from the game site. The industry experienced growth in the early-mid 2010s, and operators estimate there are approximately 50,000 escape rooms around the world (Ascalon, 2024). Escape games are immersive, with the literature describing players’ experiences of simulation in an environment that mimics real spaces (Cohen et al., 2021). These are safe environments without real-world consequences, allowing players to experience otherwise stressful scenarios in an entertaining way (Cho et al., 2023; Cohen et al., 2021).

There is a growing literature on escape rooms. Nowacki and Stasiak (2025) examine what visitors count as a memorable experience in these spaces and find that game players are drawn in most by games that are saturated with emotions, such as surprise and amusement. Dilek and Kulakoglu Dilek (2018) have examined the leisure and recreational components of these sites. These authors argue that escape room visitors seek authenticity. Pakhalov and Roxhkova (2020) find that escape rooms are popular tourism destinations and, like other tourism, museum and leisure spaces, are now incorporating more digital technology under the guise of immersion and authenticity to attract visitors and revenue (also see Kolar, 2017). The escape room literature also explores how these sites offer opportunities for education, recreation, and economic benefit (Pakhalov and Roxhkova, 2020), yet little work examines the messaging of such sites regarding social issues like imprisonment (but see Mussell et al., 2023; Stuit, 2020a).

To interrogate messaging within prison-themed escape rooms, we engage with literature on prison tourism and penal spectatorship. Scholars including Barton and Brown (2015), Wilson (2011), and Welch (2015), examine prison tourism in various case sites around the world. This work documents how prison tourism sites provide a space for safe contact with taboo phenomena like imprisonment, which draws in participants. Welch points out how museum visitors seek amusement, leisure, and escape in carceral spaces, which is ironic given that most people in real life seek to evade the pain, abuses, and neglect such spaces are notorious for. Relatedly, dark tourism scholars (e.g., Hartmann, 2014; Stone, 2018) examine sites pertaining to suffering and death such as prisons, arguing participants are drawn to them not only out of a desire to engage with the taboo, but specifically to encounter tragedy and pain experienced by othered populations. Site operators stage the authenticity of these spaces to lure participants (MacCannell, 1973) through the ‘preservation, restoration, importation and creation’ of carceral spaces (e.g., cells) and objects (e.g., restraints) (Walby and Piché, 2015), which play into their fantasies and desire for access to the real (Knudsen et al., 2016). The authenticity experience accessed via tourism and leisure sites is a manufactured facade or facsimile of actual events and experiences (also see Kleuskens et al., 2016; Luscombe et al., 2018). This literature offers some insights into why tourism is established in decommissioned sites of confinement, the way these spaces are curated, the messages communicated at these sites, and why participants visit them. Our article explores messaging in prison-themed escape room sites specifically, which lacks scholarly attention.

Prisons are marked by spaces and sounds that signal control and normalization (Marti, 2025). They exist as a site of discipline within a larger set of interconnected disciplinary institutions (Foucault, 1977). The conditions of control and normalization specific within prisons create pains of imprisonment (Sykes, 1958), notably forms of deprivation and humiliation (Hancock and Jewkes, 2011), that imprisoned people seek to evade and adapt to. The more that imprisoned people feel uncertainty and volatility behind bars, the more these pains of imprisonment can lead to a feeling of weight and tightness that erode a sense of self (Crewe, 2011). Escape rooms commodify these aspects of imprisonment and punishment.

The commodification of punishment relies on stereotypes and tropes, and as a result this process leads to distortions in public views of the realities of imprisonment and the lives of criminalized people (Wright, 2000). For Brown (2009), penal spectatorship and commodified sites of social control create a distancing of the self from a depicted or represented object of fear (e.g., the prisoner). Denham (2020) examined the commodification of criminalized bodies and the gore of transgression, arguing that repeated exposure to facsimiles leads to notoriety for some infamous prisoners, which creates a punitive brand or marketing ploy out of ‘criminal justice’ representations. In our data, bodies are often presented in generic terms so that players can step into a character, missing aspects of imprisoned experience specific to class, Indigeneity, race, gender, and sexuality, and other markers of difference. Stuit (2020a) examines forms of play in a commercial escape room organized in the Netherlands’ former Breda prison. There are many familiar scripts of imprisonment (p. 166) used in Breda's game plot, fetishizing aspects of the prison for a commercial project. While ‘the lure of the prison cell’ (Stuit 2020b: 307) and the appeal of an encounter with an authentic prison (p. 318) is used to advertise prison-themed escape rooms, the forms of play enacted by the site trivialize the realities of incarceration. Likewise, the intent at the majority of escape room sites we have investigated is to attract the most visitors possible and cater to their desires for entertainment, not the provision of education about imprisonment. From our data, one interviewee who administers Canadian heritage sites said, ‘we need to find innovative ways to get people to keep coming’, and that escape rooms ‘take some of that heaviness away from regular programming’ (interview – 5 August 2023). An interviewee that operates an American corporate-run prison escape room shared: ‘There's no message, there's no agenda, it's a haunted house. We’re just trying to entertain people at the end of the day. We want to people to be screaming but with a smile on their face, walking out there and clapping their hands’ (interview – 14 May 2024). There are few limits in terms of the sensational nature of what is depicted or the accuracies found in the content.

We use the idea of ‘carceral tropes’ to examine how ‘criminal justice’ issues are framed. Chartrand and Rougier (2021) use the idea of carceral tropes to refer to the colonial assumptions behind most penal categories and classifications. Ho (2022) uses the idea of carceral tropes to refer to the pervasive, stereotypical ideas that pervade discussions of imprisonment and punishment. Fludernik (1999, 2005) has examined carceral metaphorics and metonymics in literary texts. Oswick and colleagues (2004) argue that tropes are communicated through four main forms (metaphor, metonymy, synecdoche, and irony), although more generally tropes are any ‘figures of thought in which language is used strategically and rhetorically to set up types of relationships’ (p. 107). They argue that metaphor, metonymy (substitution of one object to refer to another unrelated thing or process, often leading to displacement from one idea to another), and synecdoche (using the part to refer to the whole or vice versa) are ‘resonance tropes’ that tend to affirm or establish a relationship, whereas irony tends to be a ‘dissonance trope’ that creates ambiguity or incongruity.^1^ The fact that the escape rooms invite game players to escape while still invoking other ideas that justify carceral power and expansion is a form of irony that creates ambiguity and incongruity in the messaging. Prisons become a setting for play in escape rooms, which would immediately seem to disrupt the common metaphor that prison is hell (Fludernik, 2005: 230). This focus on tropes provides a useful parallel to the examination of representations in the cultural study of ‘criminal justice’ communications, which often draw on the works of Stuart Hall. Even tropes appearing in seemingly innocuous discursive accounts can have serious effects on the way people understand social issues (Nelson, 1998). We argue that many prison-themed escape rooms use wrongful imprisonment and deaths behind bars as carceral tropes, communicated through metaphor, metonymy, synecdoche, and irony. There are realities to wrongful imprisonment and deaths behind bars that scholars have studied, but from a rhetoric and communication studies perspective the goal is to examine how these curations are rhetorically used as figures of thought in cultural spaces to frame issues of penality.

Building on this approach, we draw on the idea of carceral tropes to examine how tourism and leisure sites commodify aspects of imprisonment for profit and in the process distort elements of incarceration. We focus on wrongful imprisonment and death behind bars as the two elements of penality that many escape rooms feature in game design as alluring carceral tropes. The content conveyed in escape rooms is, as we reveal, full of stereotypical material about penality assembled through the clues that the game players work through. Rather than inviting reflection from participants, the focus on miscarriages of justice reproduces the notion that the punitive injustice apparatus comprised of policing, the courts, and ‘corrections’ itself is legitimate and well-functioning. We also argue that the focus on deaths behind bars ends up reproducing the idea that prisoners are conniving and/or unintelligent, and deserving of their fate. On one hand, an analysis of these displays could advance the idea that it is good that the escape rooms focus on, for example, wrongful conviction and death as fundamental elements of imprisonment, which is more than occurs at some other penal heritage sites including many prison museums (see Kleuskens et al., 2016; Luscombe et al., 2018). However, in using wrongful imprisonment and execution as carceral tropes, with the misleading and skewed elements that tropes entail, escape room sites neglect and naturalize structural and systemic forms of carceral violence, including colonial and racist violence, that allow them to persist.

## Research design

In our study of what prison-themed escape rooms communicate and fail to convey about imprisonment, as well as the degree to which they address the realities of the punitive injustice apparatus, we draw on fieldwork conducted at 31 sites visited in 2023–2024 (see Table 1). Members of our research team played escape room games at 14 sites in Canada (3 decommissioned and 11 faux-prison sites), 10 in the United States (2 decommissioned and 8 faux-prison sites), 4 in Australia (1 decommissioned and 3 faux-prison sites), and 3 in Aotearoa New Zealand (1 decommissioned and 2 faux-prison sites). We selected those settler colonial countries as they feature escape games in and outside of prisons, and have similarities in popular penal culture. Examining these cases can allow us to see how they mobilize escape games in similar or different ways. As part of this fieldwork, we interviewed 18 people involved in operating these sites. This fieldwork involved observation of game participants and staff, in addition to documenting game narratives, the way sites are organised and designed, and the objects used. In our fieldnotes, we recorded observations regarding spatial, visual, narrative, and performative arrangements at these sites. In interviews, we asked about the design and script of escape rooms, the messages operators seek to convey, and reactions among visitors. Our positionality as critical researchers situated in Canada and Aotearoa New Zealand who played these games mean our insights will differ from those of other scholars and other visitors more broadly given that the popular consumption of penality is characterized by polysemy, whereby various meanings can be formed (Walby and Piché, 2011). During site visits and interviews we carry our knowledge, experiences, and values with us. For example, during fieldwork members of our research team noticed how the sites compared to others visited in the regions, centred what we have learned from people with lived experiences in those spaces, and contextualised visits within the longer histories of those places.

**Table 1.** Prison-themed escape rooms study sample.

Game	Venue	Location	Site type	Most prominent game theme
Jailbreak	Escape HQ	Auckland, ANZ	Faux-prison	Wrongful Imprisonment
Redemption	Escape Dunedin	Dunedin, ANZ	Decommissioned gaol	Wrongful Imprisonment
Prison Bus	Escape Masters	Wellington, ANZ	Faux-prison	Evading Death Behind Bars
Mitch's Mate	Escape Cells at Adelaide Gaol	Adelaide, AUS	Decommissioned gaol	Evading Death Behind Bars
Escape from Alcatraz	The Great Escape	Melbourne, AUS	Faux-prison	Wrongful Imprisonment
Convicted: Prison Break	Adventure Escape Room	Perth, AUS	Faux-prison	Wrongful Imprisonment
Prison Break	Fox in a Box Perth	Perth, AUS	Faux-prison	Evading Death Behind Bars
Prison Break	The Locked Room	Calgary, Alberta, CAN	Faux-prison	Evading Death Behind Bars
Prison	VR CORE	Calgary, Alberta, CAN	Faux-prison (virtual reality)	Imprisonment
Historic SDG Jail Escape	Historic SDG Jail	Cornwall, Ontario, CAN	Decommissioned jail	Other (various)
Huron Historic Gaol Escape	Huron Historic Gaol	Goderich, Ontario, CAN	Decommissioned jail	Imprisonment
The Green Mile	KeyMasters Escape Rooms	Hamilton, Ontario, CAN	Faux-prison	Evading Death Behind Bars
Old Jail Escape Room	L’Orignal Old Jail	L’Orignal, Ontario, CAN	Decommissioned jail	Evading Death Behind Bars
Medieval Prison	TRAPPED	Mississauga, Ontario, CAN	Faux-prison	Evading Death Behind Bars
Death Row	Escape Manor Ottawa	Ottawa, Ontario, CAN	Faux-prison	Evading Death Behind Bars
The Prisoner (Advanced)	Roundabout Canada	Toronto, Ontario, CAN	Faux-prison	Evading Death Behind Bars
Death Row	Action 500: X-Cape	Dorval, Quebec, CAN	Faux-prison	Evading Death Behind Bars
Prison Break	Escaparium	Dorval, Quebec, CAN	Faux-prison	Wrongful Imprisonment
Asile Psychiatrique	A/Maze Escape Game	Montreal, Quebec, CAN	Faux-asylum	Other (psychiatric institution visitor)
Al-Patraz	Échappe-Toi	Montreal, Quebec, CAN	Faux-prison	Detective Investigation
Les Oubliettes du Pied-du-Courant	Échappe-Toi	Montreal, Quebec, CAN	Faux-prison	Detective Investigation
Prison Break – Wrongfully Convicted	Escape Revolution	Los Angeles, California, USA	Faux-prison	Evading Death Behind Bars
Prison Break	Red Door Escape Room	San Diego, California, USA	Faux-prison	Wrongful Imprisonment
Alcatraz Escape	Escape SF	San Francisco, California, USA	Faux-prison	Imprisonment
Final Confinement	Fox in a Box Miami	Miami, Florida, USA	Faux-prison	Evading Death Behind Bars
Prison Break	The Escape Game Orlando	Orlando, Florida, USA	Faux-prison	Wrongful Imprisonment
Prison Escape / Haunted Prison	Old Joliet Prison	Joliet, Illinois, USA	Decommissioned prison	Wrongful Imprisonment
Prison Room	PanIQ Escape Room	Chicago, Illinois, USA	Faux-prison	Wrongful Imprisonment
Escape from Death Row	Amaze Escape	Arlington, Massachusetts, USA	Decommissioned jail	Evading Death Behind Bars
Prison Break	The Escape Game Dedham	Dedham, Massachusetts, USA	Faux-prison	Imprisonment
Manhattan Rikers 1932	BrainXcape	New York City, New York, USA	Faux-prison	Imprisonment

We applied thematic analysis to locate patterns and develop themes from our field notes and interview transcriptions (Clarke and Braun, 2016). We inductively assigned and refined codes to represent reoccurring patterns in the data. Wrongful imprisonment stemming from a miscarriage of justice (*n* = 9) and evading deaths behind bars (*n* = 13) are the two most frequent themes that emerged from our coding and analysis. Further themes that we do not explore in this paper include seeking to escape imprisonment more generally (*n* = 5), detectives entering the cells to investigate prison escapes (*n* = 2), and other main storylines (*n* = 2). We then examined our data on miscarriages of justice and deaths behind bars in a second phase, looking for examples of metaphor, metonymy, synecdoche, and irony. Below, we focus on the two core issues noted above to explore how prison-themed escape rooms use these issues to commodify imprisonment. With each trope and each site, we contrast the representation communicated compared to local histories of incarceration, which reveals some of the silences and absences in these escape room representations.

## Analysis: encounters with prison-themed Escape rooms

Our analysis explores two of the most prevalent tropes arising from our fieldwork in prison-themed escape rooms: (1) wrongful imprisonment resulting from being wrongfully accused and/or convicted or being a political dissident and (2) evading death behind bars via executions, life sentences without the possibility of parole or other circumstances (e.g., an apocalypse). Escape room games often communicate that prisoners are deserving of their plight if they cannot overcome confinement. The message is that those who remain confined do not have what it takes to ‘escape’ or survive. Puzzle solving during the game becomes a metaphor for moral worth and incarcerated people are portrayed as inherently criminal. The games suggest prisoners cannot avoid coming into conflict with the law and being imprisoned, and lack the ability to navigate their circumstances once they are incarcerated to overcome a ‘criminal life’.

### Wrongfully imprisoned

The first carceral trope that we examine in these escape room representations is the notion of wrongful imprisonment. Many prison-themed escape rooms use miscarriages of justice as the arc or storyline for the game design. The escape games in our sample acknowledge that some people are wrongfully accused and/or convicted or in a few cases imprisoned for reasons that elicit sympathy (e.g., conscientious objectors in the context of war). Yet, even those people are depicted as deserving their fate if they do not figure out how to ‘escape’ on their own or with some help by escape room staff or other players. A common message in escape room marketing is ‘do you have what it takes to escape?’ (e.g., Escape Dunedin), placing responsibility on the player/prisoner for achieving freedom or failure. Imprisonment itself is treated as natural. Even in games featuring corruption among prison staff, there is no critique of incarceration per se, which is itself an ironic twist. This lack of critique was addressed in an interview with an escape room operator who noted, ‘We didn’t try to build something that deep about the justice tunnel in America’ (interview – 26 April 2024). By using wrongful imprisonment as a carceral trope, these escape rooms distract from the structural injustices that contribute to racialized mass incarceration and the pains of imprisonment for all other criminalized persons.

We start with examples of escape rooms in decommissioned carceral sites. These former carceral sites are now repurposed as commercial venues. Escape Dunedin's *Redemption* game is held in a decommissioned gaol and police station in Otago/Dunedin, New Zealand. The site was preceded by immigration barracks that were converted into temporary gaol buildings in 1855 (Heritage New Zealand, n.d.). The current building was constructed and operated as a gaol from 1898 until 2007. It served as a women's prison from 1959–1974, and then a remand and short sentence prison for men until 2007. It was also the home of the Central Police Station from 1919–1994 (Dunedin Gaol, n.d.). Māori have long been imprisoned in Dunedin including in 1869–1873 and 1879–1881 following resistance to land seizures by the Crown in Taranaki (Petchey, 2015).^2^ Since 2007, the building has been operated by Dunedin Charitable Trust (Dunedin Gaol, n.d.), who rent the site to an escape room business, Escape Dunedin. The site is also used for other purposes such as public tours and filming.

The escape room game narrative is focused on imprisonment of conscientious objectors in 1916 during the First World War when the New Zealand government introduced conscription (Escape Dunedin, n.d.). Players take on the role of citizens arrested on suspicion of pacifism and have the opportunity to escape by cooperating with imprisoned comrades in the adjacent cell. Staff roleplay as jail officers here, handcuffing players to bring them to locked cells, a metonymic reference to prison authority. The game narrative has social resonance and ‘cultural power’ (Welch, 2015: 255) in the city where a memorial commemorates conscientious objectors, including Archibald Baxter (Littlewood, 2022).^3^ The game does nod to prisoners’ experiences and history. In addition to drawing on Baxter's story, it features mugshots of real imprisoned people from the time period on the walls such as Margaret McNeill, who was a sex worker from the early twentieth century (a synecdochic reference to whole generations of people imprisoned there). The escape room seeks to introduce a sense of authenticity and the pains of imprisonment at the time by utilising items from the era such as austere furniture including a ‘guard desk, vintage rotary phone, typewriter, old lamp, and lockers’ (fieldnotes – 10 February 2024). The infrastructure at the site is largely original to 1898 and features small, dark cells.

Despite efforts to stage authenticity, the game does not communicate details about the realities of imprisonment experienced at the site, and it is up to players to understand that the game draws on real events and to educate themselves outside of the gaming experience. Audiences may find playing the part of a conscientious objector exciting as a taboo subject given that Australian and New Zealand Army Corps soldiers are highly valorized as protectors. There may also be an excitement attributed to standing up for one's convictions to the point of imprisonment. However, the focus on conscientious objectors is a somewhat jarring selection of a game theme in the context of who has been predominantly incarcerated at the site over time (e.g., people who are not conscientious objectors). The site also does not include narratives regarding contemporary imprisonment, including miscarriages of justice. In New Zealand, Te Kāhui Tātari Ture (the Criminal Cases Review Commission) is inundated with applications. Over the last decade, 893 people have had their convictions quashed or sent back to court (Osborne and Akoorie, 2023). Māori are among those cases – they are hyper-imprisoned and have comprised 50% or more of incarcerated people in New Zealand since 1980 (Te Uepū Hāpai i te Ora, 2019). Instead of delving into these realities, there is metonymy in this case as Dunedin stands in for a historical prison in general rather than a repurposed site with a specific colonial, penal history that continues to reverberate in the present through the mass imprisonment of Māori.

In the United States, there are also prison-themed escape rooms premised on the idea of wrongful imprisonment. One example is at Illinois State Penitentiary (also known as the Old Joliet Prison), which is a decommissioned prison used by Thirteenth Floor entertainment group as an escape room and haunted house in Joliet, Illinois. The prison operated from 1858 to 2002, and among the people confined there were Vietnam-draft conscientious objectors who were subject to Hepatitis experiments, one example among many human rights violations at the prison (Halpern, 2021). The Joliet Area Historical Museum (n.d.) began running tours of the site in 2018, while escape room and haunted house activities began in 2021.

The escape games, one prison-escape themed and the other asylum themed, are five-minute games with little emphasis on narrative. There is a metonymic framing here since Joliet stands in for the whole carceral institution in general. Some of the characters that appear in the attractions are drawn from the history of the prison, such as Odette Allen, the wife of a warden who died in a 1915 fire in housing attached to the prison (Lawson, 1982). Also depicted is a prisoner, Joseph Campbell, who was sentenced to death for the murder, which was commuted to a life sentence. He was a Black imprisoned man made to work as a house servant to the warden and is believed to be innocent. Yet, while the site invites visitors to ‘follow in the footsteps of the inhabitants to unravel the mysteries’ in the asylum escape room and speaks to attractions ‘decorated by the remains of failed [escape] attempts’ (Old Joliet Haunted Prison, n.d.), there is no attempt to educate visitors about those details, including more recent experiences of imprisonment. Black people continue to face injustice and are imprisoned at a rate of 7.8‒1 compared to white people in Illinois (Sentencing Project, n.d). Wrongful convictions are estimated to be as high as 15.4% of all convictions in the United States (Leo, 2005). Rather than address these injustices, the escape room and haunted house attractions are curated with fun in mind, with characters depicted as monsters and motifs drawn from decades ago. Speaking about an innocent people who died in the decommissioned prison site, one prison operator shares, ‘Since it's a haunted prison, we use the ghost as a chilling character’ and ‘midway is the vibe… and there's themed, iconic characters that interact with the guests’ (interview – 14 May 2024). This reveals an irony present across these sites insofar as injustices related to incarceration are hinted at but then players are invited to retreat into game play. Attractions sensationalize imprisonment and feature elements that did not occur in the prison, creating further distance between visitors and carceral realities, both past and present. For example, the prison-themed escape room game featured ‘a plot involving weapons and explosives’ (fieldnotes – 14 May 2024), which does not reflect local carceral realities.

Escape games in faux-prisons also invoke the idea of wrongful imprisonment. For example, to begin the *Prison Room* game at Paniq Escape Room in Chicago, the ‘ominous voice of the narrator explain[s] that the warden has information that could exonerate you on a flash drive’ in his office (fieldnotes – 26 April 2024). While he is out for lunch, you have one hour to retrieve the device and escape. An example of metonymy, the flash drive stands in for ideas of justice and due process. As an interviewee in our study noted, ‘injustices are part of the idea’ in their *Prison Break* game, ‘where motivation and excitement comes from beating the bad guy, in this case the nefarious warden, because you know you are innocent’ (interview – 14 August 2024).

Another example of metonymy is Escaparium's (n.d.) *Prison Break* held in a commercial plaza in Dorval, Quebec, near Tiohtià:ke/Montreal. Constructed within commercial business space, the game creates the illusion of a prison. Escaparium is an escape room franchise in Quebec with games rated among the best in North America by aficionados (e.g., Spira 2024). In the narrative of its *Prison Break*, players encounter the following escape or die scenario: ‘you’ve been framed for a murder you didn’t commit. They’ve been trying to get a confession out of you for months now. Even though they have already sentenced you to death, you won’t break, you didn’t do it, but no one believes you’ (fieldnotes – 28 July 2024). Players are divided between two cells, which have been fitted with an authentic-appearing door along with bars, a toilet, and a bed. Behind the toilet is a tunnel leading to a representation of a prison yard with high walls topped by barbed wire, which work as metonymic signs standing in for the broader experience of incarceration. A staff member roleplays as a prison officer and leans into a trope of corruption, giving the players an item to help them escape. Similar to the other escape rooms in our dataset, relationships with staff as well as prisoner camaraderie are essential to escaping, but also portrayed as ‘criminal’, a definite irony. This element of ‘prisoners working together to commit more crimes’ (i.e., escape) advances the narrative that the imprisoned ‘will always be problematic people’ who need fixing and are of a corrupt character, even those who are falsely convicted (fieldnotes – 28 July 2024). The element of escape is communicated as transgression and criminal, and collaboration as conspiracy. Game play makes it clear that all escape activities must be done secretly for fear of punishment. The game does not communicate that solidarity among prisoners is used by institutions and staff to facilitate aspects of prison work, while it is also controlled and disrupted as imprisoned people are viewed as untrustworthy and at the bottom of the social hierarchy (Fayter and Kilty, 2023). The game also does not share information about the prevalence and realities of wrongful conviction in Canada, including its history and the role gender, age, indigeneity, and race play in police and prosecutorial misconduct that contributes to this phenomenon (Mason, 2020).^4^

Lastly, we turn to The Great Escape's *Prison Breaks Escape from Alcatraz*, situated in a commercial plaza next to the University of Melbourne in Carlton, Victoria, Australia. Like the Dorval escape room, the operators constructed a faux-prison within a commercial business-space. The narrative involves imprisonment in Alcatraz for an offence the character did not commit where players are presented with a scenario whereby they either escape or ‘this prison [will] become your final story’ (The Great Escape, n.d.). Death behind bars is metaphorically equated with a story that one authors. The escape game is situated in small cells with toilets, books, and ‘a bench to represent a bed’, featuring walls ‘covered in a wallpaper designed to look like stone and graffiti’ (fieldnotes – 20 December 2024). Game imagery includes the American flag, the Statue of Liberty, references to US Marshals, and Western-themed wanted posters. Items do not lend to a sense of authenticity at this site and it is clearly metonymy, with these national symbols standing in for American justice and cultural identity. The depictions are sensationalist and stereotypical representations of an old prison in a different country, lacking detail about actual experiences there, and far removed from experiences in Victoria, Australia. Staff play the role of a prison officer and chide the players about a prior escape attempt and recapturing upon entry, stating: ‘back again? You won’t be able to escape this time’. Humour is used to create distance from reality, with offences such as ‘putting monopoly money in ATMs’ and ‘squirting the [American] President with a water pistol’ as reasons for imprisonment, an irony because ‘real crimes’ that people are criminalized for are often just as minor (fieldnotes – 20 December 2024). The game does not communicate about imprisonment in Victoria, where Aboriginal and Torres Strait Islanders are hyper-imprisoned at 12.3% of all prisoners (while comprising 1% of the state population)^5^ and where Pacific peoples are also imprisoned including from New Zealand, Fiji, Samoa, and Tonga (Australian Bureau of Statistics, 2022, 2024) in relation to factors connected to colonialism such as poverty, racism, and institutionalisation (Anthony and Stanley, 2024; Enari and Taula, 2022). Despite these local realities, imprisonment at The Great Escape is portrayed through a lens of escapist popular culture, drawing on representations of the most infamous prison in American movies and television, and providing reasons for imprisonment divorced from reality.

These escape rooms commodify wrongful conviction and imprisonment, which simply become a trope for game players to be amused by. The main message communicated in these games (somewhat ironically) is that if players fail to ‘escape’ a life of ‘crime’ and imprisonment, it would be due to an inability to navigate the game by solving clues, and not due to the game (or the punitive injustice apparatus) itself causing that condition and ‘failure’. In so doing, these displays ‘figuralize’ accounts of the prison setting (Fludernik, 1999: 66) and distract from the realities of why people are imprisoned in the case countries, including ongoing legacies of colonialism. The storylines of most games are individualizing. This escape theme ends up depicting those who can escape wrongful imprisonment and those who do not as deserving their fate owing to individual capacities, while narratives about structural forces that could foster a deeper reflection about the injustices at work in the name of ‘criminal justice’ and the need for an escape from criminalization and punishment are a curious absence.

### Evading death behind bars

Evading death behind bars, most commonly via executions or imprisonment for life without parole, is a second carceral trope used by several prison-themed escape room games. Executions are perhaps the most iconic element of penality, with the electric chair most often representing the power of death that the carceral sublime can inflict on those behind bars (Martschukat, 2002). Execution devices are metaphors for state power. There are two tendencies in our dataset in terms of the latent meanings in these displays. First, this predominant focus on execution detracts from the pains of imprisonment and the many other forms of death behind bars including those owing to a lack of access to medical care (Scallan et al., 2019), violence from prison officers (McClelland et al., 2024), as well as suicide (Horii, 1994), especially for racialized people. Second, by using the execution storyline in an escape room, these representations advance the notion that the prisoners trying to evade the pains of imprisonment and death at the hands of the state are conniving and trying to cheat the system, rather than abiding by the state's judgement and using formal tools of appeal.

Adelaide Gaol's *Mitch's Mate* escape game is held in the decommissioned Adelaide Gaol in Tarntanya/Adelaide, South Australia. The gaol operated from 1840 to 1988 (Langmead, 1997) and approximately 300,000 people were imprisoned throughout this period. Forty-five people were executed there (Adelaide Gaol, n.d.). After its decommissioning, the South Australian Department for Environment and Heritage reopened the site as a museum and tourist attraction. Game players are locked in an old cell with a prison bed, shelf full of books, nightstand, and other vintage props to add authenticity. The cell is original to the prison – small, made from stone, with a heavy metal door facing an outdoor courtyard. The game is set in 1978–1979 and players try to escape following the clues of a friend who was previously imprisoned there. The game is not grounded in reality – for example, the cell is full of items that belonged to ‘Mitch’, which facilitate your escape when in practice those would have been cleared away. Following gameplay, players can self-tour the prison site. The displays are extensive, with numerous signs describing elements of the site including how the architecture evolved over the decades, along with the ‘colourful characters’ who ran it (e.g., prison governor William Baker Ashon) and were imprisoned there (e.g., Henry Dawson who worked as a clerk while imprisoned for burglary) (fieldnotes – 22 September 2024). Such depictions, however, do not fully reflect life in the gaol (see Agutter, 2014), nor speak at all about the continuing realities of imprisonment in Australia. There is no information about the stolen land and imprisonment of Aboriginal and Torres Strait Islander peoples. Yet graffiti and artwork on the prison walls, including the Aboriginal flag, portraiture of an Aboriginal man, and a landscape with dotwork and an emu with no contextualisation indicate that Aboriginal peoples were imprisoned there. The prison displays emphasize the executions that took place at the gaol, provide details regarding hanging protocols, equipment and staff who were involved, and allow visitors to step foot directly in the spaces executions occurred and where people are buried (fieldnotes – 22 September 2024). There is no mention of the murder of Aboriginal peoples, who were not executed at the prison but at the scene of their ‘crimes’ to edify communities and reinforce colonial rule (Anderson, 2015).^6^ Aboriginal people were also executed without any guise of legal process, such as during the Maria Massacre in 1840 (Anderson, 2015). In the decades since capital punishment was abolished, Aboriginal and Torres Strait Islanders continue to face death. There have been 590 deaths behind bars since the Royal Commission into Aboriginal Deaths in Custody 34 years ago (Australian Institute of Criminology, 2025). Most deaths in custody are due to systemic neglect including inadequate or withheld medical care (Walsh, 2022).

While capital punishment has been abolished in Canada since 1976, executions are among the most prevalent themes in Canadian prison-themed escape room sites. One example can be found at the Ancienne Prison de L’Orignal (L’Orignal Old Jail), which is a decommissioned prison in L’Orignal, Ontario, Canada. The oldest jail in Ontario and second oldest in Canada, it was built in 1825 and operated as a small francophone jail until 1998. During this time, more than 500 prisoners were incarcerated there, including those living with mental illness but without convictions (Brown, 2006). Five men were executed by hanging. Following its decommissioning, the second floor was occupied by a courthouse, which continues to operate today (L'Orignal Old Jail, n.d). The site is run by the United Counties of Prescott-Russell and was opened to the public in 2007 for exhibits and tours (Walby and Piché, 2015), and more recently escape games.

The *Old Jail Escape Room* game in L’Orignal begins with players locked in two cell blocks, which are linked by a narrow passageway along the back wall. The cells feature no windows, and are small and ‘coffin-like’. The narrative involves prisoners seeking to escape as they have been left to die. In a dystopian and apocalyptic plot twist, the jail officers have abandoned the facility since ‘the outside world is crumbling’. Fitting this narrative, no staff participate in the game, unlike most other prison-themed escape rooms where members of our research team conducted fieldwork. As part of game-play wanted posters are used, but these depict fictional characters. There is no discussion of specific prisoner's stories or experiences of life and death in the jail during game-play. However, the site is open for tours in French or in English after game-play where players can gain more insights about imprisonment. There are exhibits featuring narratives of police officers, lawyers, and former jail staff, but ‘no specific prisoner's stories’ (fieldnotes – 5 August 2023). Visitors can also access the yard where a reconstructed-hanging platform stands as a means of showing what the site looked like during the hangings of five men who were executed there (Piché et al., 2017), a metonymic device referring to capital punishment. Not addressed in the game or elsewhere onsite is the reality that deaths behind bars continue, including due to neglect by staff. This includes the death of Justin St Amour who died in hospital after he hung himself while on suicide watch at the Ottawa-Carleton Detention Centre about an hour west of L’Orignal (Doyle et al., 2021). Between January 2000 and December 2024, at least 2324 people died behind bars in Canada (McClelland et al., 2024).

The prospect of death is a key idea communicated in a number of sites. For instance, Escape Masters’ (n.d.) offers a *Prison Bus* escape game in a high-rise building in downtown Pōneke/Wellington, New Zealand. This is a commercial space where a faux-prison space has been constructed. Escape Masters is a franchise with another location in Auckland. The story involves players being transported to ‘spend the rest of your life in prison’ without parole at Wellington Maximum Security (fieldnotes – 29 November 2024). It is unclear whether the site being referenced is Rimutaka or Arohata Prison, or divorced from actual prisons in the region. The *Prison Bus* escape room begins in a simulated prison transport vehicle. The bus features grey seats, ‘empty walls’, columns of lockers at the front, prison jumpsuits, and barred windows. The rear window is painted with ‘a scene of a desert road with cacti and yellow sand’, likely referencing American pop culture tropes about imprisonment, since there is no ‘view anywhere in New Zealand’ like it (fieldnotes – 29 November 2024). Once players escape the bus they enter an outdoor area that simulates an exercise yard with faux brick walls, a wooden bench, exercise weights, and a basketball hoop. The next room is a warden's office featuring an array of officer hats and real photos of prison officers at graduations and in front of Parliament. While prison officers are memorialized in a positive light, the context of imprisonment in which they work is ignored. There have been 201 deaths in custody between 2015 and 2024, of which 127 have been labelled as natural and 74 as unnatural (Department of Corrections New Zealand, n.d.). The many deaths in custody – despite the abolition of capital punishment in New Zealand in 1989 and only one life sentence without parole on record since that time – is not information one learns in the game.

Fox in a Box's *Final Confinement* situated in a commercial plaza in downtown Miami, Florida is another prison-themed escape game that is oriented around the theme of death. The escape game takes place in a faux-prison constructed within a commercial business space. The central narrative is that players face a life sentence at a maximum security prison where ‘you can no longer appeal your case, your fate has been sealed. Your only way out seems to be in a body bag’ (Fox in a Box Miami, n.d.). The body bag is a grim metonymic device referring to death in prison as a form of escape. A staff member plays the role of a prison officer in riot gear and tells the players they are headed for the electric chair. Alongside the gallows, the electric chair is among the most iconic element of prison sites in the United States (Martschukat, 2002), which is invoked metaphorically to refer to state power. Players are given a uniform to wear (a t-shirt labelled with ‘property of state penitentiary’ and ‘maximum security inmate’) and are divided between two cells that face each other. The cells have a metal bench, handcuffs, lockers, toilets, a wall-mounted phone, and a bed that all appear ‘old and authentic’ (fieldnotes – 13 August 2024). Participants must collaborate using clues from each other's cells to escape. Before getting out of the first set of cells players are transported to a new room and locked in cells as they are told a riot broke out in the prison. Those cells connect to another room that represents a prison officer's control room with surveillance equipment. The posters on the wall and items are Second World War-era or mid-century coded, and there are ‘several items donated by retired NYPD’ and ephemera evocative of Jamesville Penitentiary located in Syracuse, New York including a photo of the prison, a map of Onondaga County, prison staff jackets, and a flag of the New York State coat of arms (fieldnotes – 13 August 2024). In marketing the game, the operators advertise that the bars are from an original prison – Jamesville Penitentiary – making an appeal to authenticity to lure tourists to the site for a staged encounter with a genuine carceral artefact. Jamesville Penitentiary operated from 1899 until 1983 when it was replaced by another facility (Onondaga County Sheriff's Office, n.d.). Capital punishment was declared unconstitutional in New York in 2004 yet is still legal in Florida. While *Final Confinement* players engage in game where losing results in a fictional death, lost is the reality that there have been over 400 deaths reported in Florida custody in each of the past five fiscal years from 2020–2021 to 2024–2025 alone, in addition to executions (Florida Department of Corrections, n.d.a, n.d.b).

There are also some games that feature dissonant, if not incoherent, displays of death and execution. At Roundabout Canada in Toronto, *The Prisoner* game incorporates fake blood splatters all around the escape room that are not connected to the game narrative. At KeyMasters Escape Rooms in Hamilton, we encounter a mock electric chair in *The Green Mile* that game players are asked to sit in, although it is not connected to the flow of the game either. As was observed by a research team member, ‘This was the only room which actually had a prop version of an electric chair that participants have to sit on. This also served to increase the pressure on players as I believe this would be disturbing even for people who do not study imprisonment’ (fieldnotes – 18 February 2024). At the end of the *Prison Break* game in The Locked Room in Calgary there is another replica of an electric chair. However, it is not a prisoner strapped in. Instead, as a research team member documented, ‘we jolted in surprise and shock when seeing a mannequin of a correctional guard who had visibly died on the electric chair’ (fieldnotes – 26 July 2024). Placing a prison officer in an electric chair runs counter to not only what one would expect from most carceral storylines, it is also a scene not directly connected to the game narrative. Suffice it to say that the carceral tropes appearing in prison-themed escape room games are not always as coherent as those found in literary texts (see Fludernik, 2005).

Overall, we observed that these escape rooms commonly commodify execution and death by imprisonment via life without the possibility of parole as a carceral trope. The displays distract from the many other more common forms of death in prisons and jails including neglect, lack of access to medical care and violence from staff. These escape games also communicate that those who can evade death at the hands of the state through escape are conniving by skirting formal mechanisms, which reproduces dominant societal stereotypes about prisoners, while commodifying aspects of the experience and systems of incarceration.

## Discussion

As Welch (2015) noted in relation to prison museums, these forms of penal popular culture invite escape *to* rather than *from* the prison and similar sites of confinement and brutality. Stuit (2020a) makes a similar argument about the escape room organized in the former Breda prison, where game players can see the prison from the inside. We see the same tension in the incarceration-themed escape rooms examined here. The game player is invited to a room often adorned with faux or contrived carceral objects, sights and sounds, meant to signal fear and provide the allure of an encounter with the authentic. Yet the focus is on play and entertainment. There are objects (some claimed to be authentic) that the game players touch to feel like they are getting closer to imprisonment, selected for ‘their capacity to produce meaning’ (Welch, 2015: 5). The escape room designers further add the narrative layers of wrongful imprisonment and death behind bars as carceral tropes for the visitor to work through. However, the invitation to play and escape is predicated on an actual distancing of the self (a fleeing) from a depicted or represented object of fear (the imprisoned person) in the safe, sanitized and stereotype-laden game zone (also see Brown, 2009). Such social distancing is manifested through games in Canada, Australia, Aotearoa New Zealand, and the United States often drawing on American carceral tropes and narratives detached from the actual place the games take place in. Beyond gesturing to the violence work wardens or prison officers engage in, there is also no engagement with critical ideas such as the abolition of imprisonment or an analysis of penal states at these sites. Rather than challenging prisons, escape room sites treat them as natural and necessary, celebrating them as places where ‘the bad’ and ‘the incompetent’ get their due.

Escape rooms are entertainment zones where tourists seek a semblance of authenticity and a heavy dose of hedonism (Dilek and Kulakoglu Dilek, 2018; Kolar, 2017). Some punishment scholars might be unconcerned with the content of prison tourism sites due to a trend in academia to treat matters of popular culture as irrelevant or mere forms of amusement without real effects in the world (but see Fiske, 2011 on why popular culture deserves investigation). Perhaps the fact that escape rooms foreground escape as a dominant feature seems rather banal. However, the way escape and imprisonment is communicated in these games in particular ways matters.

Our inquiry does point to the importance of understanding how forms of play (e.g., escape rooms, videos games, etc.) are related to penality in the twenty-first century. As Huizinga (1955) argued in an early contribution to cultural studies, forms of play are relevant to discussions of law, state social control, and war insofar as play can be a point of genesis for forms of culture. In Western rational thinking, ‘play is the direct opposite of seriousness’ (p. 5), yet it is only from a serious analysis of the kinds of culture that play gives rise to that we can understand the institutions that govern our lives.

Building on this line of argumentation, Bogost (2010) contends that games and play are not mere forms of amusement, rather the contents invite players to take on particular worldviews while they engage in gaming. Stuit (2020b) makes the argument that the escape game organized in the former Breda prison offers different, sometimes diverging themes, however none of them lead to educative or contemplative forms of play. Examining the career of panopticism from Bentham's conceptualization, to Foucault's inquiries, to its resurfacing ‘as an architectural mould in prison design’, Fludernik (2017: 20) likewise traces out how carceral metaphorics and metonymics can feed into realities of control. The forms of play in the carceral escape room contribute to and reflect the culture of control that is emerging in the age of online media consumption. Is it any wonder that we are seeing the rise of prison-themed escape rooms in this era of celebrity prisoners, social media accounts from inside, and reality prison television?

Our findings reflect this line of argumentation by suggesting that the content and effect of prison-themed popular culture, including incarceration-themed escape room games, is important to study, as it can play a role in shaping public views of the punitive injustice apparatus, and in reproducing it. Such sites minimize or trivialize the pains of imprisonment, inverting the sense of liminality associated with prison metaphors. Whereas most prison metaphors convey a sense of liminality as a life-or-death issue (Fludernik, 1999: 65), the liminality and transgression of escape that game players are invited to experience is reduced to the difference between winning or losing. Such sites also render prisons as a place of fun and escapism for spectators, and naturalize them as a necessary institution for people who seemingly belong there. Playing the role of the wrongly imprisoned, ‘a victimized prisoner who is really just a regular person infers the existence of another kind of prisoner – the deserving, guilty, antagonizer’ (fieldnotes – 26 April 2024). This is consistently the case across the four countries in our study.

## Conclusion

Escape room sites around the world are designing prison-themed games for tourists and patrons seeking a good time. Though they are popular forms of tourism and leisure, we have shown how these sites commodify aspects of imprisonment and punishment. We used the idea of carceral tropes to examine the way prison-themed escape rooms across Canada, the United States, Australia and Aotearoa New Zealand reproduce dominant stereotypes about imprisonment and prisoners. The idea of carceral tropes draws attention to the way metaphor, synecdoche, metonymy, and irony operate as cues in cultural sites to convey assumptions about imprisonment and punishment. We acknowledge that the curators of these sites might be constructing these carceral tropes less strategically and more as a habit, what Pavlich (2018) calls habits of criminalization and law and order thinking.

Though escape rooms may seem detached from operational sites of imprisonment, our work examines how penal ideologies and practices are upheld in part through tropes conveyed in popular culture such as in escape rooms. These escape rooms are devoid of historical context and silent on carceral violence against Indigenous peoples across Canada, the United States, Australia, and Aotearoa New Zealand. These sites commodify the bodies and suffering of prisoners. Indeed, we have argued that the escape room prison as spectacle obscures material experiences of incarceration, especially those of Indigenous peoples. Such representations of penality not only deserve academic inquiry but are inherently political (Wright, 2000). Any hope for social change or a world without carceral power requires studying and contesting stereotypical representations of punishment. The fact that escape rooms are also used for education and training in some settings means they could certainly be more educational and less stereotypical. Escape rooms might seem like an odd target for anti-carceral knowledge generation and organizing. Still, they are part of the landscape of culture and communication that supports what Foucault (1977) conceptualized as the carceral archipelago, understood as a network of carceral control within and beyond traditional prisons (also see Welch, 2015). Such forums of penal popular culture, where representations shape public views of the punitive injustice apparatus, ought to be among the sites where struggle towards de-centring imprisonment as a way of responding to transgression happens.

## Notes

1. On the issue of metonymy versus metaphor, Fludernik (2005: 236) argues that metonymy is often a property or element of a broader metaphor discourse, and that metaphor and metonymy can be entangled when used in literary texts and other communications. Valverde (2006: 22) argues ‘if metonymy can be visualized as a horizontal connection between signs that are not logically related, but only placed together, metaphor can be visualized, by contrast, as a vertical relationship between a sign that one can plainly see and another sign that is not actually visible’.

2. This included arrests of more than 400 peaceful protestors near Parihaka and the Waimate Plains in 1869, with some dying while imprisoned. In 1881, the government invaded and destroyed the pacifist settlement in Parihaka, arresting and imprisoning its leaders. Indigenous peoples in New Zealand, as well as Canada, Australia, and the USA, are hyper-imprisoned through neo-colonial institutions, laws, and policies (George et al., 2020).

3. Baxter, along with 13 other conscientious objectors, were transported to the frontlines in France and subject to Field Punishment No. 1, which involved being bound to a post in a crucifixion pose (Archibald Baxter Memorial Trust, n.d).

4. Indigenous peoples are hyper-incarcerated in Canada and represented among wrongful convictions cases. The incarceration rate of Indigenous persons was 8.9 times higher than for non-Indigenous persons in 2020/2021 (Statistics Canada, 2023). This hyper-imprisonment is normalised, despite occurring amidst structural violence, and cultural and territorial dispossession (Arbel, 2019).

5. Aboriginal and Torres Strait Islanders are hyper-imprisoned across Australia, and accounted for 36% of all prisoners across Australia in 2024 while comprising 3% of the population. The most common serious offences Aboriginal and Torres Strait Islanders are convicted of in Victoria are unlawful entry with intent and acts intended to cause injury (Australian Bureau of Statistics, 2024). Blagg and colleagues (2021) present insights from Elders on violence in northern Australia. Elders link violence to colonialism including alcohol, trauma, and jealousy, and solutions must be rooted in communities and centre Elder's knowledge. Further, at least 71 people have been found wrongfully convicted between 1922 and 2015 in Australia (Dioso-Villa, 2015), and Aboriginal and Torres Strait Islanders are represented in this number for reasons such as racist stereotypes, pressures to plead guilty, language and translation difficulties, and inadequate legal representation (Roach, 2015; Stratton and Sigamoney, 2020).

6. It was not until 1972 that the right of a judge to sentence an Aboriginal person to a public hanging was revoked (Criminal Law Consolidation Amendment Act 1971 SA). Capital punishment was abolished in South Australia in 1977 (Lennan and Williams, 2012).
